# A Low Temperature Limit for Life on Earth

**DOI:** 10.1371/journal.pone.0066207

**Published:** 2013-06-19

**Authors:** Andrew Clarke, G. John Morris, Fernanda Fonseca, Benjamin J. Murray, Elizabeth Acton, Hannah C. Price

**Affiliations:** 1 British Antarctic Survey, High Cross, Madingley Road, Cambridge, United Kingdom; 2 School of Environmental Sciences, University of East Anglia, Norwich, United Kingdom; 3 Asymptote Ltd, St John’s Innovation Centre, Cambridge, United Kingdom; 4 Institut National de la Recherche Agronomique, Thiverval Grignon, France; 5 School of Earth and Environment, University of Leeds, Leeds, United Kingdom; University of Waterloo, Canada

## Abstract

There is no generally accepted value for the lower temperature limit for life on Earth. We present empirical evidence that free-living microbial cells cooling in the presence of external ice will undergo freeze-induced desiccation and a glass transition (vitrification) at a temperature between −10°C and −26°C. In contrast to intracellular freezing, vitrification does not result in death and cells may survive very low temperatures once vitrified. The high internal viscosity following vitrification means that diffusion of oxygen and metabolites is slowed to such an extent that cellular metabolism ceases. The temperature range for intracellular vitrification makes this a process of fundamental ecological significance for free-living microbes. It is only where extracellular ice is not present that cells can continue to metabolise below these temperatures, and water droplets in clouds provide an important example of such a habitat. In multicellular organisms the cells are isolated from ice in the environment, and the major factor dictating how they respond to low temperature is the physical state of the extracellular fluid. Where this fluid freezes, then the cells will dehydrate and vitrify in a manner analogous to free-living microbes. Where the extracellular fluid undercools then cells can continue to metabolise, albeit slowly, to temperatures below the vitrification temperature of free-living microbes. Evidence suggests that these cells do also eventually vitrify, but at lower temperatures that may be below −50°C. Since cells must return to a fluid state to resume metabolism and complete their life cycle, and ice is almost universally present in environments at sub-zero temperatures, we propose that the vitrification temperature represents a general lower thermal limit to life on Earth, though its precise value differs between unicellular (typically above −20°C) and multicellular organisms (typically below −20°C). Few multicellular organisms can, however, complete their life cycle at temperatures below ∼−2°C.

## Introduction

Biologists have long been interested in the physical limits to life, particularly in relation to life in extreme environments on Earth and the possibility of life elsewhere in the universe. Whereas the high temperature limit to cell division is fairly well defined [1], the lower limits for life are essentially unknown [2].

The survival of bacteria and unicellular eukaryotes has been demonstrated down to −18°C in unfrozen hypersaline polar lakes [3]–[5], but in these extreme environments active cell division is typically confined to summer when shallow waters are warmed and illuminated by the sun. Whilst photosynthesis has been reported in the Antarctic lichen *Umbilicaria aprina* at −17°C [6], spoilage of frozen food by the yeast *Rhodotolura glutinis* at −18°C [7] and metabolism of bacteria from permafrost at −20°C [8], [9], the lower temperature limit for cell growth and division remains unclear [2].

Here we present theoretical arguments and empirical evidence which clarify the biophysical constraints that determine the lower temperature limit for life on earth.

## Theoretical Background: Life at Low Temperatures

Philosophers and scientists have struggled to achieve a universally accepted definition of life [10]. In considering life at extremes, however, a critical distinction is that between metabolism and replication (reproduction), as emphasised by von Neumann [11]. An essential feature of life is completion of the life cycle; for a bacterium or archaean this means cell division to produce daughter cells, for a sexually reproducing eukaryote it means completion of the cycle from zygote to zygote.

**Figure 1:**
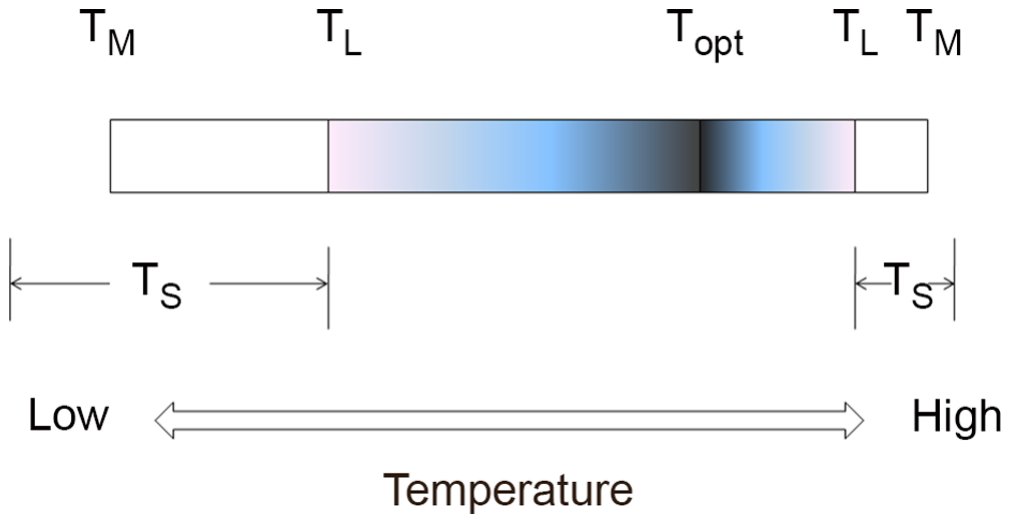
Temperature limits for life. T_L_: temperature limit for completion of the life cycle, T_M_: temperature limit for metabolism, T_S_: temperature limit for survival. Note that T_S_ can be above, at, or below T_M_. The shaded area shows temperature range over which the organism can complete its life cycle, and the white areas show the temperature range (upper and lower) for survival. T_opt_ is the temperature at which growth rate is maximal, which is typically closer to the upper T_L_ than the lower T_L_.

An organism may, of course, continue to metabolise even when it is unable to complete its life cycle. We can therefore draw an important distinction between two different threshold temperatures: a threshold for completion of the life cycle, T_L_, and a threshold for metabolism, T_M_ (Figure 1). Between T_L_ and T_M_, the organism is viable and metabolising, but unable to complete its life cycle; the difference between the lower bounds T_L_ and T_M_ is especially important in extreme low-temperature environments. A third important threshold is that for survival, T_S_, although this does not exhibit a consistent relationship with T_L_ or T_M_: the lower T_S_ may be at T_L_, at T_M_ or below T_M_. Thus in a chilling-sensitive organism, a critical physiological system fails at a relatively high temperature, and T_S_, T_L_ and T_M_ may even coincide. By contrast, in a cold-hardy organism, T_S_ may be below T_M_. Between T_M_ and T_S_ the organism is in a state of suspended animation, but can resume metabolism once the temperature increases and re-crosses the T_M_ threshold. An important difference between the high and low temperature limits for life is that at high temperatures T_S_ is always reached; in contrast at low temperatures an organism may never reach its T_S_, even when taken close to absolute zero [12]. The upper and lower T_L_ thresholds thus mark the limits to life on Earth, as existence outside these boundaries does not allow for completion of the life cycle.

**Figure 2:**
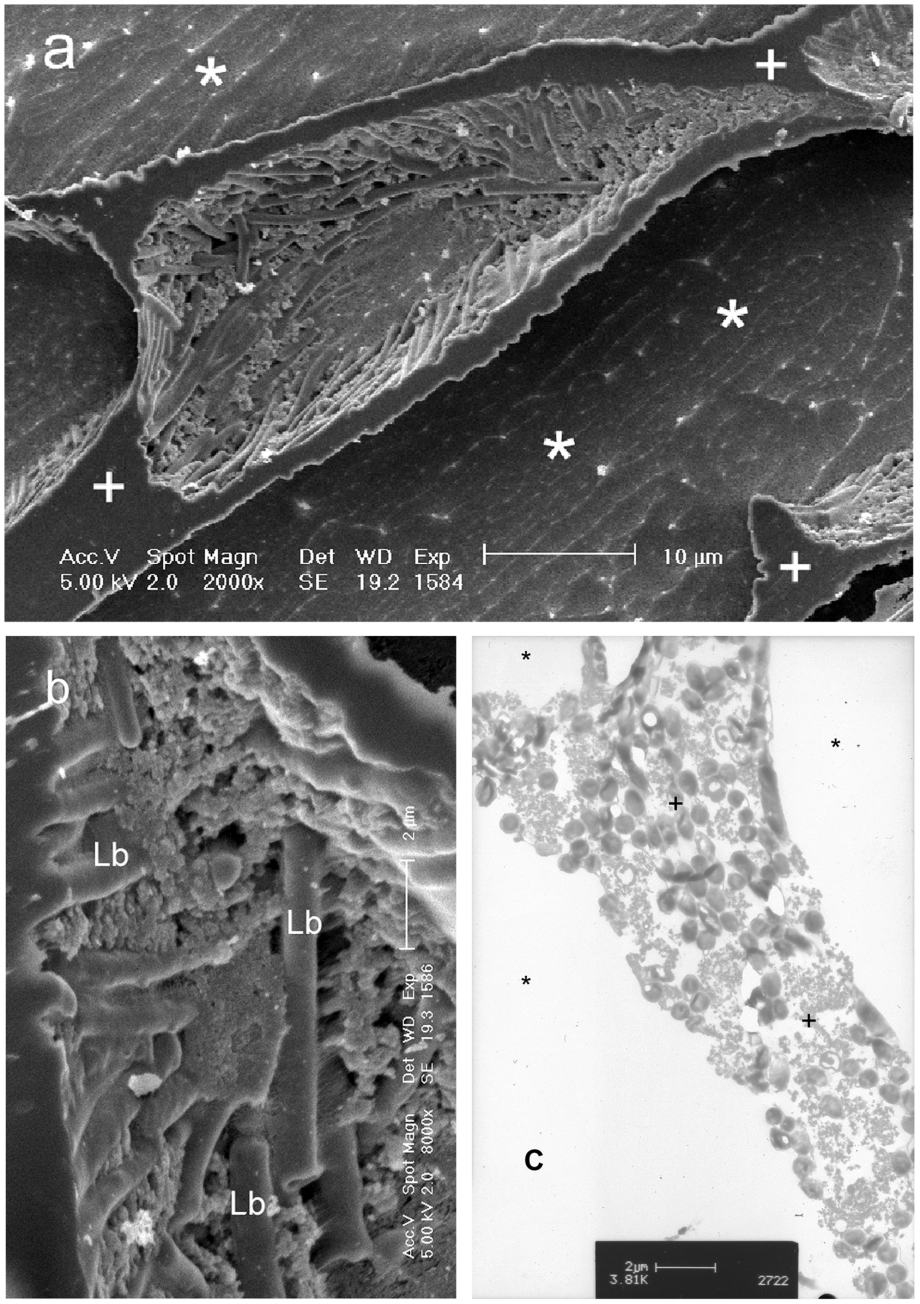
Structure of ice and cells following cooling of a cell suspension. Cryo scanning electron microscopy of fracture samples of *Lactobacillus delbrueckii* ssp. *bulgaricus* following slow cooling. A. Low power image of a fractured sample, showing cells confined to the unfrozen liquid between ice crystals; this unfrozen matrix is highly freeze-concentrated as solutes are excluded from the growing ice crystal. B. High power image of the interface between ice and the freeze concentrated matrix, with bacterial cells labelled. C. Freeze substitution of the same sample reveals cells packed within the freeze concentrated matrix. In all samples the spaces originally occupied by ice crystals are revealed as voids following sublimation of ice. The labels mark ice voids (*) *Lactobacillus* cells (Lb) and freeze concentrated solute (+).

Cells at low temperatures must cope with the reduced molecular kinetic energy of the environment and the consequent lower rate of many physical processes. In addition there are stresses arising from reduced membrane fluidity, changes in intracellular pH, and loss of macromolecular integrity [13], [14]. Additional challenges arise when ice is present in the environment immediately external to the cell. Following ice nucleation, solutes are rejected from the growing ice crystal and are concentrated in the remaining liquid; importantly, microorganisms will also be excluded from the ice lattice [15] (Figure 2). Cells may be damaged in the presence of extracellular ice through hydraulic, osmotic or solute toxicity mechanisms. In addition, intracellular ice can form when the cooling rate is sufficiently high that the cell cannot maintain osmotic equilibrium with the environment [16]. Intracellular ice formation generally requires faster cooling than is typical of the natural environment. For example in bacteria, intracellular ice only forms when cells are cooled rapidly (>100 K min^−1^) in dilute aqueous media [17], [18], and in the yeast *Saccharomyces cerevisiae* intracellular ice formation requires cooling rates faster than 20 K min^−1^ [19]. Intracellular ice is observed only rarely in the natural environment [20], although freezing of extracellular fluids does occur in some multicellular plants and animals living in seasonally cold climates [21]–[23].

Vitrification (also known as the glass transition) occurs when a liquid begins to behave as a solid during cooling, but without any substantial change in molecular arrangement or thermodynamic state variables [24]. As temperature decreases, all molecular motions, translational and internal, become progressively slower until a critical temperature is reached where there is insufficient energy for significant translational or rotational molecular motion to take place over a meaningful timescale. This is the vitrification or glass transition temperature, Tg, and it is defined operationally as the temperature at which viscosity exceeds 10^12^ Pa.s [24]. Thermodynamic state variables (pressure, volume, internal energy, entropy) change minimally during the glass transition: vitrification thus does not involve the entropy change and associated exotherm associated with freezing [25]. There are, however, changes in thermodynamic response variables such as heat capacity and thermal expansivity, and the glass transition can be detected by differential scanning calorimetry (DSC) from this change in heat capacity.

Intracellular vitrification is more complex than in simple bulk liquids. This is principally because the interior of the cell is extremely crowded [26], approximating a colloid in physical structure. As colloids dehydrate they exhibit a sharp increase in viscosity and undergo a colloid glass transition [27]. Cellular dehydration, whether associated with a shift to anhydrobiosis or withdrawal of water driven by freeze concentration of the extracellular medium (see Figure S1), will thus induce vitrification of the cell interior. Intracellular vitrification is primarily the result of this dehydration and is analogous to the vitrification of a colloid, rather than the glass transition in bulk water. The low rates of cooling characteristic of the natural environment coupled with the dehydration of the intracellular milieu driven by freeze concentration of the extracellular medium means that outside the laboratory vitrification of the cell contents will almost always occur before intracellular ice can form, and is likely to be a process of widespread ecological significance for free-living unicells.

Vitrification has however not been widely reported from the natural environment, probably because it is technically difficult to demonstrate. It can, however, be detected with a sensitive DSC from the variation in heat flow associated with the change in heat capacity at the glass transition. We have investigated the incidence of vitrification in a range of unicellular organisms. The aim of the study was to determine the incidence of vitrification in unicellular organisms cooled slowly in the presence of extracellular ice.

## Materials and Methods

Intracellular glass transition temperature, Tg, was determined with differential scanning calorimetry (Diamond, Perkin Elmer LLC, Norwalk, CT, USA, equipped with a liquid nitrogen cooling accessory, CryoFill, Perkin Elmer). The cell types selected comprised five bacteria: *Lactobacillus delbrueckii* ssp. *bulgaricus*, *Pseudomonas syringae*, *Corynebacterium variabile*, *Arthrobacter arilaitensis* and *Streptococcus thermophilus*, two photosynthetic eukaryotes: *Auxenochlorella protothecoides* and *Chlamydomonas nivalis*, and two heterotrophic eukaryotes: *Debaryomyces hansenii* and *Saccharomyces cerevisiae* (for cell lines see Table S1). After washing cells with peptone water (1 g L^−1^), about 10–50 mg of each cell pellet was scanned following cooling to −100°C and heating to 20°C at 10 K min^−1^. Cell concentrations were typically 10^8^–10^10^ ml^−1^. The protocol for temperature calibration and determination of the glass transition temperatures is described elsewhere [28]. Cell suspensions were examined in the frozen state using cryo scanning electron microscopy (CryoSEM) and freeze substitution, as described previously [18], [28].

Cells were also examined by Raman spectroscopy on a humidity controlled microscope stage, as the Raman spectrum allows unambiguous identification of chemical compounds and phase changes [29]. Cell suspensions were mounted on a silanised glass cover slip, which has been shown previously not to catalyse nucleation. The chamber was constructed of Teflon and sealed on the base with an O-ring. The sample was viewed through a window on top of the chamber. The humidity in the nitrogen flowing over the cells was controlled by passing nitrogen gas through a bubbler immersed in a temperature controlled bath and diluted using mass flow controllers (MKS 1179) to produce the desired humidity. The humidity of the gas downstream of the cell was recorded with a dew/frost point hygrometer (General Eastern D-2 sensor). The temperature of the stage was set using a re-circulating chiller and the temperature of the sample was measured with a platinum resistance thermometer embedded in the stage immediately below the sample. The experiments were performed with a 514 nm laser coupled to a Renishaw inVia Raman microscope system equipped with a Leica microscope and Olympus 20× long working distance objective. The experimental protocol is described in detail in [30].

To provide an environmental context for the vitrification study we also modelled the thermal properties of water droplets of the sizes found in clouds and likely to effect dispersal of microbial cells. The modelling assumed homogeneous freezing to be a stochastic process. Parameterisations to describe the temperature dependence of the water activity of the liquid at the ice-liquid equilibrium line and the dependence of the nucleation rate coefficient with the shift in water activity from the ice-liquid equilibrium line were taken from [31], and we modelled the water activity at which we expect 50% of a population of droplets of a given size in a given time to freeze. The method is described in detail elsewhere [28]. The thermal histories within droplets of water of various sizes following ice nucleation at different degrees of undercooling were modelled on the assumption that initial ice formation is isenthalpic [32].

## Results

**Figure 3:**
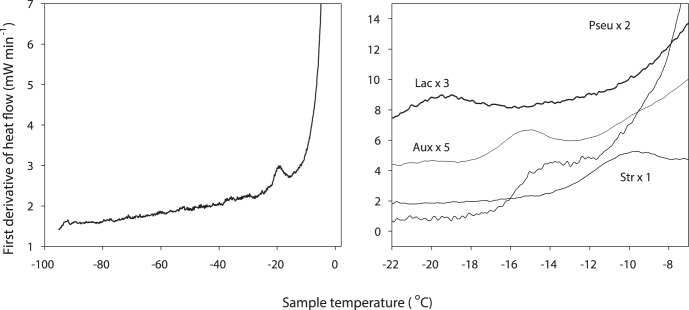
Differential scanning calorimetry (DSC) traces showing vitrification in cell suspensions. A (left panel). DSC trace of *Lactobacillus delbrueckii* ssp. *bulgaricus* which clearly shows a vitrification (colloid glass transition) temperature, Tg, of −19.3°C in samples that have been cooled below −90°C and then warmed. The glass transition during warming is indicated by the deviation in the first derivative of the heat flow, associated with a change in heat capacity as the bacterial cells devitrify at Tg. The large increase in heat flow at temperatures above Tg corresponds to the freezing of the aqueous medium in which the cells were suspended. B (right panel). Composite of DSC traces from four organisms (three bacteria, and an alga). The traces have been scaled as shown to render them visible on a single ordinate scale.

Using sensitive DSC, we have measured the intracellular glass transition (vitrification) temperature, Tg, in a range of cell types when cooled slowly in the presence of extracellular ice. Under the experimental conditions used, any intracellular freezing would have been detected by the release of latent heat associated with the change in entropy that accompanies the phase transition from liquid water to ice in the cell during cooling, or the reverse during warming; none was seen. Vitrification was detected by the variation in heat flow associated with the change in heat capacity, together with the absence of any release of latent heat (Figure 3). No vitrification signal was seen in control runs (medium without cells) (see Figure S2). The signal detected in the sample runs was the glass transition temperature of the maximally freeze-concentrated intracellular compartment (conventionally designated Tg’). Any glass transition events in the extracellular compartment would be expected to be at a lower temperature, but were not detected in this study because the volume of extracellular medium was so low in relation to that of the cells (Figure 2) as to render any Tg signal undetectable.

In all cell types examined, intracellular vitrification occurred at high sub-zero temperatures (Table 1), with no sign of intracellular freezing. All cell types are viable after vitrification under these experimental conditions (Morris, unpublished data). The observed values of Tg ranged from just below −10°C in thermophilic species and the ice-nucleating bacterium *Pseudomonas syringiae*, to around −25°C in three species, including the snow alga *Chlamydomonas nivalis*. These temperatures are well above typical environmental temperatures for high latitudes in winter, indicating that intracellular vitrification must be a process of widespread ecological relevance for free-living unicells.

**Table 1:** Intracellular vitrification temperature for a range of free-living microorganisms.

Organism	Tg (°C)
Eubacteria	
* Lactobacillus delbrueckii* ssp. *bulgaricus*	−19.3 (0.9)
* Pseudomonas syringae*	−13.9 (0.9)
* Corynebacterium variabile*	−25.6 (0.6)
* Arthrobacter arilaitensis*	−26.0 (0.8), −21.0 (0.8)
* Streptococcus thermophilus*	−11.6 (1.0)
Photosynthetic eukaryotes	
* Auxenochlorella protothecoides*	−15.1 (0.7)
* Chlamydomonas nivalis*	−24.2 (0.8)
Heteroptrophic eukaryotes	
* Debaryomyces hansenii*	−11.6 (1.0)
* Saccharomyces cerevisiae*	−12.3 (1.1)

Intracellular vitrification temperature (Tg) determined by DSC. Tg is the mean of 3–4 independent measurements, with the typical SD of the measurements being 0.8°C. Note that in *Arthrobacter* two vitrification peaks were evident in the DSC traces. Standard deviation from three replicate DSC runs shown in parentheses.

Vitrified algal cells were examined by Raman spectroscopy on a humidity controlled microscope stage. Control samples of sucrose solution exhibited a clear Raman water peak that responded to external relative humidity, but this response ceased once the solution vitrified at low relative humidity. In contrast, the vitrified cells appeared to be completely unresponsive to relative humidity, and the Raman signal indicated that these cells contained little or no bulk water.

## Discussion

The data presented here are, to our knowledge, the first direct evidence of intracellular vitrification in a cell suspension under ecologically relevant conditions, that is in a dilute aqueous medium and the absence of external cryoprotectants. Vitrification was observed in all cell types examined, and intracellular freezing was observed in none. The data show clearly that during slow cooling in the presence of extracellular ice, free-living microbial cells will be subject to dehydration as freeze-concentration of the external medium withdraws water from the cell, and at a temperature above ∼ −26°C the cell contents will undergo a colloid glass transition. Following rapid rates of cooling (>20 K min^−1^) intracellular ice formation can be detected by DSC in the yeast *S. cerevisiae* [19], but we did not detect any intracellular ice at the slow rates of cooling employed in this study. The rates of cooling required to induce intracellular freezing are generally only experienced by cells in certain specialised habitats such as leaf surfaces (see Table S2, Figure S3).

Chilling-sensitive species do not vitrify, for as the temperature falls they reach their T_S_ and die before ice forms in their environment. For cells to vitrify they must remain osmotically active at subzero temperatures. The importance of this is exemplified by species of the unicellular alga *Chlamydonomas*: whereas the temperate *Chlamydomonas reinhardtii* is intolerant of cooling to low temperatures, the snow alga *C. nivalis* is able to withstand repeated episodes of freeze-thaw in its environment and remains osmotically active at subzero temperatures. These physiological differences are associated with differences in membrane composition, structure and function [33], [34].

In cells that remain osmotically active when ice is present in their immediate external environment a critical factor driving an intracellular colloidal glass transition is the presence of proteins and other macromolecular structures. As water is withdrawn from the cell interior, driven by freeze concentration of the extracellular medium, its internal water activity decreases (see Figure S1). If the cell contents consisted purely of an aqueous solution of protein, then the Tg would be approximately −10°C [35], [36]. However the cell also contains a large number of small molecules, including some which may be synthesised to act as cryoprotectants. As pure aqueous solutions these small molecules typically have a Tg below −50°C at ice-water equilibrium; it is the mixture of proteins and small molecules which dictates that cells undergo intracellular vitrification at intermediate subzero temperatures [36]. The vitrification temperature will thus vary somewhat with the precise composition of the internal cell environment, and this may allow Tg to be adjusted to match ecological circumstances (for example by varying the level of small cryoprotectant molecules). Such variation in Tg is, however, likely to be constrained by viable compositions of the intracellular milieu, as is shown by the Tg values observed (a range of ∼15 K: Table 1).

The second key factor which determines that a cell will vitrify at low water activity is the densely packed interior of the cell [26]. The colloidal nature of the intracellular milieu means that as water is removed, the increased packing slows intracellular relaxation processes. There is a sharp increase in intracellular viscosity and the cell undergoes a colloid glass transition [27]. The vitrification of the cell interior of a free-living microbial cell exposed to ice in the external environment is thus primarily the result of dehydration and is analogous to the vitrification of a colloid rather than the glass transition of bulk water.

Although our data indicate that vitrification is inevitable for any free-living microbial cell cooled in the presence of external ice, the range of Tg values found in this study (Table 1) indicates that the exact value of Tg varies, presumably in response to changes in the balance between macromolecules (predominantly proteins) and small molecular weight compounds, including cryoprotectants. The relatively high value for Tg found in the ice-nucleating bacterium *Pseudomonas syringae* and the low value found in the snow alga *Chlamydomonas nivalis* suggest that Tg may be adjusted, within bounds, in response to the particular ecology of the individual organism. An organism such as *C. nivalis* would presumably gain a selective advantage from a low Tg that allowed it to continue metabolism to a relatively low temperature, since ice is usually always present in its natural habitat.

Once vitrification has taken place, the high viscosity of the intracellular medium means that diffusion of oxygen and metabolites effectively stops [25]. Under these conditions metabolism must cease. These cells, however, remain viable and can return to metabolism and growth once they have warmed above their Tg. The importance of vitrification to the survival of cells at low temperature is that the glass transition does not involve the change in density that accompanies nucleation and freezing. A cell that freezes is subject to enormous mechanical stresses, which are frequently lethal. A cell that vitrifies is also behaving effectively as a solid, but retains its internal structure and integrity. It is this maintenance of internal structure that is the key factor in allowing a return to viability once an increase in environmental temperature drives the resumption of molecular movement and hence metabolism [12]. There is remarkably little difference between vitrified and living cells in terms of bulk structure, composition and thermodynamic state variables [37]. The difference comes in the extent of molecular motion, small-scale variations in entropy within the cell, and the flux of energy.

These observations also have implications for cell recovery following cryopreservation and freeze drying. It is usually assumed that cells dehydrate as a function of the increasing concentration of the extracellular medium until the latter vitrifies [19]. However our data demonstrate that at slow rates of cooling intracellular vitrification will occur at high sub−zero temperatures, and thus often before the extracellular medium itself vitrifies. At temperatures below Tg cells will be osmotically unresponsive as the extracellular concentration continues to increase and cells will not respond osmotically during warming until the intracellular environment has returned to the fluid state. Small molecular weight cryoprotectants would be expected to modify intracellular Tg, and this might be a factor in their mode of protection.

### Microbial Metabolism in Glacial Environments

The rapidly developing interest in the possibility of life in deep subglacial lakes or on bodies such as Mars or Europa has focussed considerable attention on life in glacial environments. Microbial cell division has been reported to continue at subzero temperatures, though it is likely that cell doubling times at these temperatures may be very long [9]. Current data suggest that microbial growth may extend to −20°C, but there are no convincing demonstrations of growth below this temperature [38]. A practical demonstration of the importance of this threshold is that despite a century of refrigeration technology, there are no recorded cases of spoilage organisms which grow below −20°C [39]. Taken together, these observations suggest that whilst a few organisms may be able to depress their Tg as low as −25°C (Table 1), a more general value would be −20°C.

In environments where ice is present but the temperature is above Tg, osmotically active cells continue to metabolise, albeit at a slow rate. A classic early demonstration of this was the discovery of microbial growth in unpasteurised ice cream at −10°C [40]. Examples of such habitats from the natural environment include cryoconite holes on glacier surfaces [41] and high latitude saline lakes, though in these environments growth and cell division typically only proceed when temperatures increase (for example in surface waters during summer) [3]–[5].

**Figure 4:**
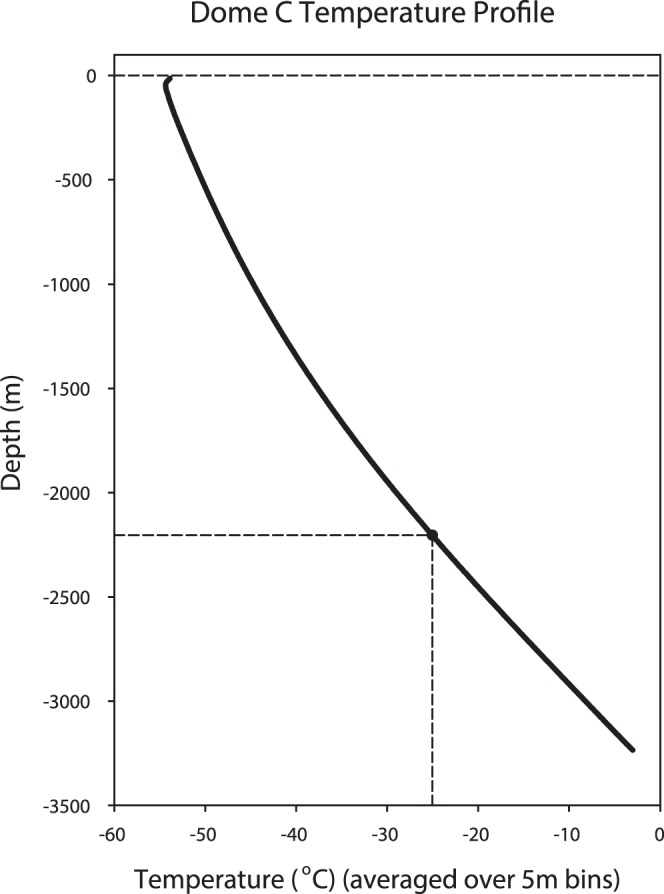
Temperature profile through an ice core taken at Dome C, Antarctica. Data are averaged over bins of 5 m. The dotted lines show a putative threshold depth above which cells will be vitrified and hence not metabolising, and below which cells may be in a fluid state and able to metabolise, albeit slowly. At the bottom of the ice core the temperature may be sufficient for cell growth and division to be possible, assuming the presence of sufficient free energy (suitable electron donors and acceptors) and a source of nutrients. Core temperature data courtesy of the European Project for Ice Coring in Antarctica, EPICA.

A wide variety of microbial cells has also been reported from glacial ice [42], [43]. Our data imply that microbial cells retained in glacial ice at temperatures below −25°C (that is, below Tg) must be vitrified. Metabolism has, however, been proposed for microbial cells in glacial ice, albeit at very slow rates, on the basis of anomalously high concentrations of methane [38] and nitrous oxide [44]. Temperature data from ice cores suggest that throughout much of the upper depths of continental ice caps, temperature is below Tg and hence cells will be vitrified. It is only towards the base of the ice sheet, where temperatures are higher because of the upward transfer of geothermal heat, that temperatures are sufficiently high for cells to be above their Tg (Figure 4). Any discussion of putative biological activity within glacial ice has to confront the difficulty of distinguishing metabolism from purely geochemical activity. The unfrozen water between ice crystals surrounding the cell will have solutes in multimolar concentrations, the pH will be highly modified and there may be extracellular enzymes present. Under these conditions, chemical kinetics are greatly modified; many reactions are accelerated and new reaction products may be generated which can include apparent metabolic products [45]. An example of the difficulties of interpreting empirical data comes from permafrost, where release of CO_2_ has been reported down to −39°C [46]. Rates of CO_2_ release below −20°C were very low, though they were above the background rate for sterile (autoclaved) soil. However whereas the isotopic signature (^14^C/^12^C) of the released CO_2_ decreased with incubation temperature down to −20°C, it remained invariant below this temperature. This shift in isotope dynamics suggests that the CO_2_ release observed below−20°C may have come from geochemical processes rather than metabolism.

### Microbial Metabolism, Survival and Dispersal in Clouds

There is one natural environment where water undercools extensively and there is no ice in the system; this is in clouds, where water droplets can remain liquid down to temperatures approaching −40°C (the homogeneous nucleation temperature for pure water [47]). In this environment any microbial cells present may continue to metabolise, albeit very slowly. This is possible because in the absence of freeze-induced dehydration the internal cell contents to not undergo vitrification. Cloud droplets thus offer a natural habitat, possibly the only such habitat on Earth, where microbial cells can continue to metabolise at temperatures below those at which in other environments they would normally vitrify [48]. They may even be able to undergo cell division [49], [50]. The ecological importance of this is that viable microbial cells within clouds can be dispersed globally [51].

**Figure 5:**
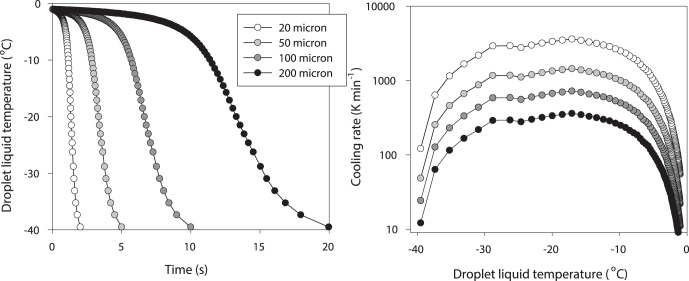
Temperature following nucleation of water droplets of different sizes. A (left panel). Temperature of cloud droplets following ice nucleation at an environmental temperature of −40°C modelled for droplets of diameter 20 µm (left hand trace), 50 µm, 100 µm and 200 µm (right hand trace). The traces show the cooling phase after release of latent heat has raised droplet temperature from −40°C to almost 0°C. For all droplets cooling rates exceed 100 K min^−1^ and for the smaller sizes exceed 1000 K min^−1^. B (right panel). Cooling rate (K min^−1^, log scale) for these droplets, showing the very fast cooling rates following nucleation at −40°C in small droplets; these rates are sufficient to induce lethal intracellular freezing in any cells contained within the droplet.

Cells in undercooled cloud droplets are, however, extremely susceptible to being killed by intracellular ice formation should the droplet nucleate. Once nucleation has been initiated, droplet temperature will first rise to its melting temperature as latent heat is released; this will be followed by extremely rapid cooling as the ice droplet achieves thermal equilibrium with the atmospheric environment (Figure 5a). The latter may involve rates of cooling up to 1000 K min^−1^ (Figure 5b); at such cooling rates any microbial cell within the droplet will be killed through intracellular ice nucleation [17], [18].

This fate is avoided if the droplet nucleates at high sub-zero temperatures when it is only slightly undercooled, as any microbial cells it contains will then undergo freeze-concentration dehydration and vitrify. This may even be induced by the bacterial cells themselves, mediated through ice-nucleating proteins on the external surface [47], [52]. Should this happen then the droplet will freeze at a relatively high sub-zero temperature, leading to freeze-concentration of the extracellular water and osmotic dehydration of any microbial cells in the droplet. Under these circumstances the cell contents will vitrify rather than freeze, and the microbial cells would be expected to survive further cooling (note the relatively high value for Tg in the ice-nucleating bacterium *Pseudomonas syringae*: Table 1). This emphasises the survival value for airborne bacteria of initiating nucleation, and offers a simple explanation for the high levels of viable bacteria with ice-nucleating activity that can be isolated from snow and hailstones [53]–[57]. The likelihood of intracellular freezing among microbial cells without ice-nucleating activity in cloud droplets will also constrain the ability of such cells to be dispersed globally.

There have been a number of reports that microorganisms isolated from polar environments contain intracellular antifreeze proteins [58]. It has been argued that the function of these compounds is to protect the cell from lethal injury during cooling by preventing intracellular freezing. These compounds are, however, typically identified as antifreezes on the basis of their activity *in vitro*; in most cases we do not know their function *in vivo* [59]. Moreover, outside the laboratory intracellular freezing is rare, usually requiring cooling rates faster than typical of the natural environment, and is almost always lethal. Our data indicate that under natural rates of cooling, and in the presence of extracellular ice, free-living microbial cells will vitrify and intracellular ice formation is not a normal event. This poses the evolutionary question of why such cells should have an antifreeze. One speculative possibility is that their antifreeze activity may be important in reducing the chances of potentially fatal ice nucleation during warming from the vitrified state. It is also possible that these proteins have other physiological functions and their antifreeze activity is simply coincidental. The interesting question is thus whether proteins with incidental antifreeze activity are also to be found in cells where subzero temperatures are never encountered.

### Implications for Multicellular Organisms

We have demonstrated vitrification in a range of free-living unicellular organisms (Table 1). These cells all remained osmotically responsive at subzero temperatures and all underwent a glass transition; in addition all the cell lines studies are viable following rewarming from the vitrification protocol used in this study (Morris, unpublished data). There is no evidence that microbial and multicellular organisms differ significantly in their intracellular composition, and so we can assume that the fundamental thermal behaviour of cells from multicellular organisms will be similar to that of free-living microbes. When ice is present in the environment immediately external to the cells, the cells will dehydrate as an osmotic response to the freeze-concentration of the remaining fluid and once temperature falls to Tg the cells will undergo a colloid glass transition (vitrification).

A key difference for multicellular organisms is, however, that the environment immediately external to the cells is a fluid whose composition is controlled by the organism. The important factor that determines whether a cell in a multicellular organism vitrifies is the presence or absence of ice in the extracellular fluid. For a multicellular organism, the presence of ice in the external environment (that is, outside the organism) is relevant only in that it has the potential to inoculate freezing within the organism.

Intracellular vitrification has long been recognised as a survival mechanism for desiccation-tolerant organisms, for seeds of plants and resting stages of other organisms [60]. Our results suggest that vitrification will also occur in the cells of those cold-hardy plants and animals where the extracellular fluids freeze at a high sub-zero temperature. Should this occur it will lead to a freeze concentration of the unfrozen extracellular fluid, dehydration of the cell interior, and a colloid glass-transition in a manner directly analogous to that demonstrated here for free-living microbial organisms.

A clear demonstration of the ecological importance of vitrification in multicellular organisms comes from studies of polar nematodes. Nematodes in general are highly tolerant of dehydration and their internal water content can change rapidly in response to external conditions. In seven species of nematode isolated from the maritime Antarctic, survival was zero when worms were suspended in oil and cooled at 1 K min^−1^. Under these experimental conditions all seven species froze internally, with median freezing points between −7.7 and −9.9°C [61]. When the same species were suspended in water and cooled at the same rate, the water droplet froze between −3 and −6°C, but nematode survival was generally good (>70% in five species, <40% in the other two). Our results suggest that the nematodes suspended in water would have undergone freeze-induced dehydration as the water droplet froze, and a colloid glass transition. The vitrified nematodes showed generally good survival, whereas the frozen nematodes did not. Freeze/thaw cycles are frequent in the environment where these nematodes live, so vitrification is important for survival. Furthermore, polar nematodes can survive very long periods in the vitrified state [62].

In the Alaskan tenebrionid beetle *Upis ceramboides* the extracellular fluids freeze at a high sub-zero temperature, and once frozen in the natural environment this species can survive down to −60°C [63]. Laboratory studies have shown, however, that survival from freezing is extremely sensitive to cooling rate [63]. Our results for free-living microbes suggest that the freezing of the extracellular fluid will dehydrate the cells and promote a colloid glass transition, and it is possible that the threshold cooling rate for survival is dictated by the dynamics of osmotic adjustment by the cells. When cooling is sufficiently slow to allow osmotic adjustment, cells dehydrate and vitrify; when cooling is too fast cells are unable to adjust and the result is lethal intracellular freezing. Since the capacity for osmotic adjustment depends on factors such as membrane composition and the density of aquaporins, we would expect the threshold cooling rate for survival to vary between species.

Many organisms living in seasonally cold environments avoid freeze-induced desiccation and vitrification of tissues through undercooling of the extracellular fluids. In insects this undercooling allows winter activity, or colonisation of very cold habitats: adults of a wingless chironomid of the genus *Diamesa* have been reported as active down to −19°C, though the larvae develop at higher temperatures in running water [64]. Insects that move into subnivean habitats may even reproduce in winter [22], though this is only at relatively high sub-zero temperatures, and well above the vitrification temperatures reported here for free-living microbial cells. In many invertebrates that avoid freezing of extracellular fluids, the presence of high concentrations of small molecular weight cryoprotectant compounds means that cells may continue to metabolise down to very low temperatures. Eventually, however, even these cells may be expected to vitrify, and recent studies have revealed such vitrification with Tg down to −58°C [65]. In the Alaskan beetle *Cucujus clavipes*, some individuals exhibit two vitrification events, suggestive of two separate compartments each with a different Tg [65]. It is tempting to speculate that this may represent the vitrification of the extracellular fluids and the intracellular matrix as separate events. These insect larvae survive vitrification, even when overwintering in direct contact with ice [65].

The fundamental biophysics of freeze-concentration and consequent colloid glass transition must apply also to plant cells, although their different ionic composition together with the presence of chloroplasts, vacuoles and starch granules mean that thermal thresholds may differ from animal and bacterial cells. Our results for free-living microbes suggest that vitrification will also occur in the cells of cold-hardy plants where the extracellular fluids freeze. Where these fluids undercool, the vitrification temperature will be lowered, but, as observed with insects [65], a glass transition will still eventually take place as cooling continues. Vitrification has been demonstrated in cortical cells of winter-hardened *Populus balsamifera* [66], and suggested to be widespread among northern trees [67] and cold-hardy woody plants [23], [68]. Interestingly, plants also show multiple vitrification events, suggesting that different fluid compartments undergo a glass transition at different temperatures, depending on their water content [66].

Organisms tolerant of extreme dehydration, such as nematodes, tardigrades or rotifers may lose their cell water through desiccation at temperatures well above zero. The resultant low water activity of the intracellular environment means that these cells will vitrify in essentially the same manner as with freeze-concentration desiccation [27]. In both cases vitrification is driven by the dehydration of the cell interior. Vitrification has been reported from desiccated stages of multicellular organisms such as rotifers, tardigrades and the crustacean *Artemia* [69]–[71]. Here, vitrification is again driven by the removal of water and the consequent colloid glass transition. This desiccation-induced vitrification can occur at temperature well above 0°C, though the vitrified organisms may then be resistant to subsequent exposure to very low temperatures [12]. Vitrification thus provides a mechanistic explanation for the similarity of the physiological effects of two environmental factors which have long been recognised as related, namely desiccation and the freezing of the extracellular environment [72].

Plants subject to dehydration water stress synthesise a range of proteins (late embryogenesis abundant proteins, LEAs, and dehydrins) that appear to be critical in maintaining cellular structure in the dehydrated state [73], [74]. These proteins have now been recorded from a range of animal phyla [75], suggesting that, together with small molecular weight cryoprotectants, they may play an important general role in maintaining cellular structure during vitrification, such that metabolism can resume in a controlled manner once the cell rehydrates.

### Concluding Remarks

On the surface of the earth, ice will invariably be present once the environmental temperature falls below 0°C [72], [76]. We conclude that intracellular vitrification is a process of universal ecological significance for free-living microbial organisms living in uniformly or seasonally cold environments. In contrast, intracellular ice formation is not. We posit that under most normal environmental circumstances, where low temperatures are accompanied by the presence of ice, any osmotically active free-living microbial cell will undergo a glass transition, and hence regulated metabolism will cease, at temperatures between about −10°C and −25°C. The data for microbial growth at low temperatures [38], [42] and the temperature sensitivity of food spoilage [39] suggest that the general lower limit is about −20°C.

Some multicellular organisms may induce their extracellular fluids to freeze at relatively high sub-zero temperatures, and we propose that this will drive a similar vitrification of their cells. Others may promote undercooling of their extracellular fluids. In these cases, cells may continue to metabolise down to lower temperatures, but evidence from both plants and insects indicates that eventually vitrification again takes place. Since organisms must return to the fluid state to resume metabolism and complete their life cycle, vitrification thus represents an operational (and absolute) lower temperature threshold for life on Earth.

Our data point to an interesting asymmetry in the high and low temperature limits for life on Earth. Eukaryotes are able to grow at temperatures up to ∼60°C [77], whereas the upper thermal limits for cell division in bacteria and archaea are considerably higher than this [1]. The general lower thermal limit for growth in free-living microbial cells appears to be ∼−20°C [38]. Whilst adult multicellular organisms on land can remain active to very low temperatures, the limit for completion of the life cycle appears to be ∼0°C [64], [78]. In the sea, a wide range of invertebrates and fish complete their life cycles at temperatures down to the equilibrium freezing point of seawater [79], which is −1.96°C. However the ability of multicellular organisms to regulate their internal body fluids means that some terrestrial species living in permanently or seasonally cold environments can allow these fluids to undercool, and hence their cells to metabolise, down to temperatures well below the lower thermal limit for growth in free-living microbes. Endotherms (mammals and birds) can regulate the temperature of their interior such that at least one species, the Emperor Penguin, *Aptenodytes forsteri*, can complete its live cycle at temperatures below −40°C in the depths of the Antarctic winter [80].

Undercooling is not an option for unicellular organisms, where ice is almost invariably present in the environment once the temperature drops below 0°C. Nevertheless there are reports in the literature of apparent microbial metabolism at temperatures well below −20°C (discussed above). We therefore suggest that any proposal for metabolising cells under these conditions must meet a minimal set of criteria to distinguish true metabolism from geochemical processes that mimic metabolism. A possible set of criteria might include some or all of:

Utilisation of an electron donor

Reduction of an electron acceptor

Maintenance of membrane potential and protonmotive force

Synthesis of ATP

Transport of metabolites across the cell membrane

Membrane viability

Ribosomal viability.

This is a tough set of criteria, but the theoretical arguments elaborated above and our experimental data indicate clearly that the burden of proof lies with the demonstration of microbial metabolism below −20°C. Unless these criteria can be met, we must assume that what is being measured is concentrated solution chemistry and not integrated metabolism.

Our results also imply that for any cells with an intracellular milieu similar to that of cells on Earth the window of opportunity for metabolism to proceed in the present climate on Mars is very limited indeed [81]. It would, however, seem perfectly possible, given sufficient free energy, for cells to exist and metabolise in the water hypothesised to exist below the surface ice layer on Europa [82]. Furthermore, our results suggest that microbial cells could feasibly be transferred across interplanetary space were they to be transported in water with sufficient solutes to induce dehydration of the cells through freeze concentration, and thereby promote vitrification. Once vitrified, cells could withstand the very low temperatures (<10 K) encountered in interplanetary space.

## Supporting Information

Figure S1 A (left panel). The equilibrium relationship between ice and water activity (a_W_) of the solution in contact with ice for a range of sub-zero temperatures (solid symbols). Also shown is the calculated homogeneous nucleation temperature of bacterial cells (1 µm diameter) in osmotic equilibrium with ice (open symbols). Note how as a_w_ is lowered, the homogenous nucleation temperature of the cells decreases to very low temperatures. B (right panel). The relationship between a_w_ and temperature of an equilibrium ice-water mixture, showing that the limit to microbial growth at a_w_ of ∼0.8 is equivalent to a temperature of ∼ −20°C.
(TIF)

Figure S2 A (left panel). Comparison of control DSC trace (peptone water only) with sample run (*Lactobacillus delbrueckii* ssp. *bulgaricus* in peptone water), showing a vitrification signal in the sample run but none in the control (no cells) run. Data are the first derivative of heat flow (mW min^−1^) and the broad vitrification peak is produced by the change in specific heat at vitrification. Both traces were taken when warming from below −90°C and show a strong endotherm trace as the suspending medium thaws. B (right panel). Comparison of DSC traces of control samples (peptone water with no cells) during cooling to below −90°C and subsequent warming. Note the strong freezing exotherm during cooling, the melting endotherm during warming and the absence of any vitrification signal.
(TIF)

Figure S3 **Frequency histograms of rates of environmental temperature change.** A (left panel). Conservative estimates based on mean hourly data. B (right panel). Less conservative estimates, based on maximum and minimum temperatures observed in each hour.
(TIF)

Table S1 Cell lines used in this study.
(DOCX)

Table S2 Summary statistics for rates of environmental temperature change (K hour^−1^).
(DOCX)
