# Supplementary figures and images for: A Low Temperature Limit for Life on Earth
Source: PLoS One. 2013 Jun 19;8(6):e66207. doi: 10.1371/journal.pone.0066207 (PMC3686811; doi:10.1371/journal.pone.0066207)

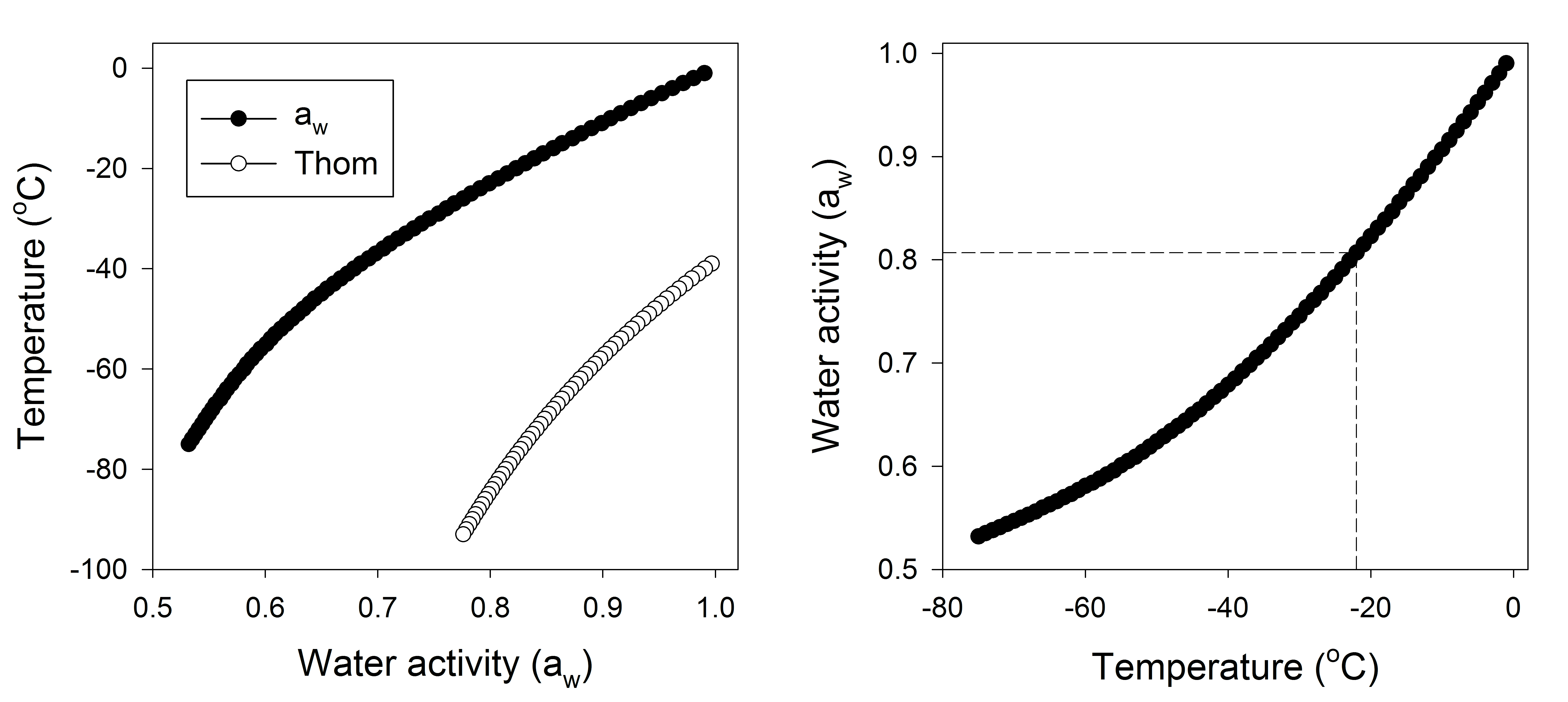

Supplement: Figure S1 — A (left panel). The equilibrium relationship between ice and water activity (aW) of the solution in contact with ice for a range of sub-zero temperatures (solid symbols). Also shown is the calculated homogeneous nucleation temperature of bacterial cells (1 µm diameter) in osmotic equilibrium with ice (open symbols). Note how as aw is lowered, the homogenous nucleation temperature of the cells decreases to very low temperatures. B (right panel). The relationship between aw and temperature of an equilibrium ice-water mixture, showing that the limit to microbial growth at aw of ∼0.8 is equivalent to a temperature of ∼ −20°C. (TIF) [file pone.0066207.s001.tif]

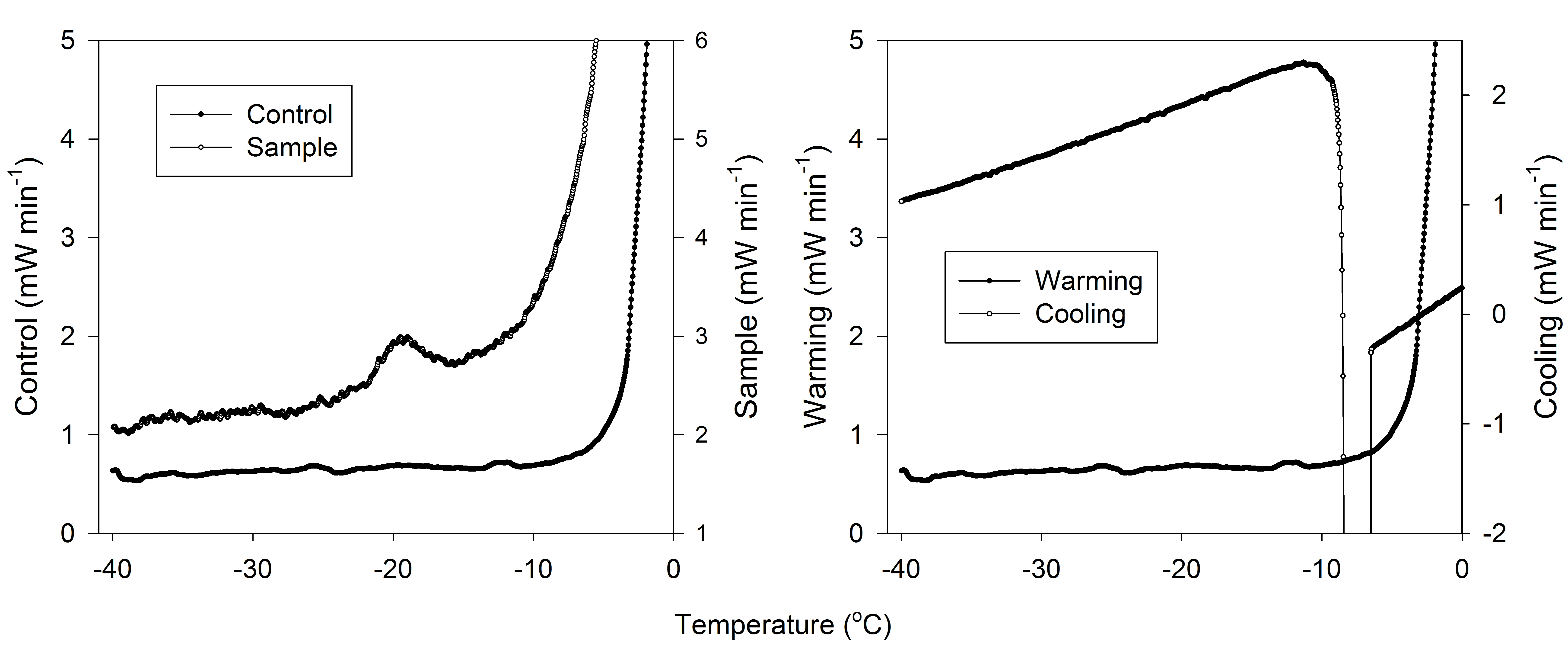

Supplement: Figure S2 — A (left panel). Comparison of control DSC trace (peptone water only) with sample run (Lactobacillus delbrueckii ssp. bulgaricus in peptone water), showing a vitrification signal in the sample run but none in the control (no cells) run. Data are the first derivative of heat flow (mW min−1) and the broad vitrification peak is produced by the change in specific heat at vitrification. Both traces were taken when warming from below −90°C and show a strong endotherm trace as the suspending medium thaws. B (right panel). Comparison of DSC traces of control samples (peptone water with no cells) during cooling to below −90°C and subsequent warming. Note the strong freezing exotherm during cooling, the melting endotherm during warming and the absence of any vitrification signal. (TIF) [file pone.0066207.s002.tif]

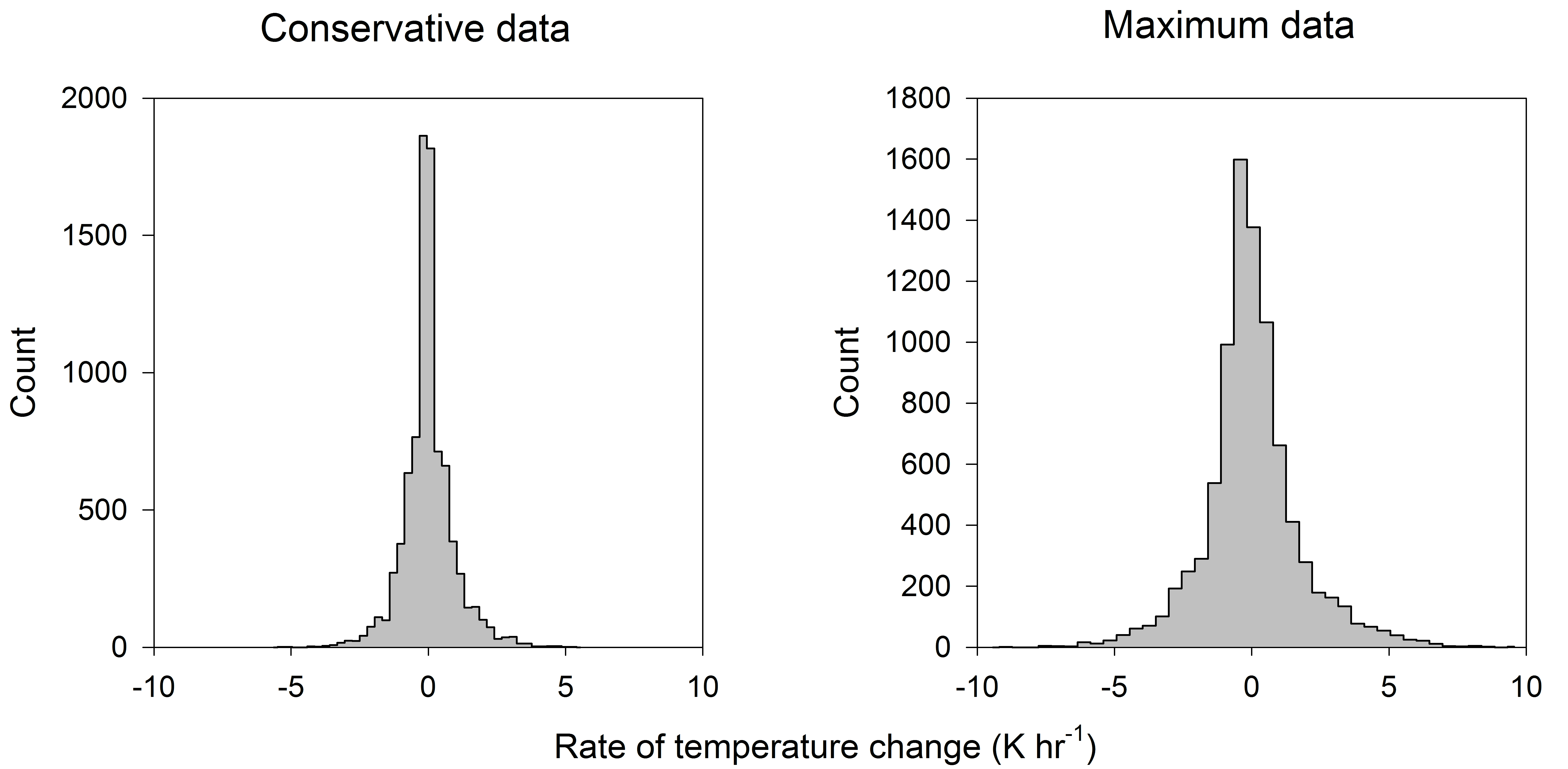

Supplement: Figure S3 — Frequency histograms of rates of environmental temperature change. A (left panel). Conservative estimates based on mean hourly data. B (right panel). Less conservative estimates, based on maximum and minimum temperatures observed in each hour. (TIF) [file pone.0066207.s003.tif]
