# Supplementary material for: A Low Temperature Limit for Life on Earth
Source: PLoS One. 2013 Jun 19;8(6):e66207. doi: 10.1371/journal.pone.0066207 (PMC3686811; doi:10.1371/journal.pone.0066207)
Supplement: Table S1 — Cell lines used in this study. (DOCX) [file pone.0066207.s004.docx]

| **Organism** | | Cell line |
| --- | --- | --- |
| Bacteria | |  |
|  | *Lactobacillus delbrueckii* ssp. *bulgaricus* | CFL1, CIRM |
|  | *Pseudomonas syringiae* | Snowmax |
|  | *Corynebacterium variabile* | 5310, DPC |
|  | *Arthrobacter arilaitensis* | Mu107, CIRM |
|  | *Streptococcus thermophilus* | CFS2, CIRM |
| Photosynthetic eukaryotes | |  |
|  | *Auxenochlorella protothecoides* | 211/7A, CCAP |
|  | *Chlamydomonas nivalis* | 11/128, CCAP |
| Heterotrophic eukaryotes | |  |
|  | *Debaryomyces hansenii* | DH34, GMPA, INRA |
|  | *Saccharomyces cerevisiae* | 338, CLIB |

CIRM: Centre International de Ressources Microbiennes, Rennes, France

DPC: Dairy Products Research centre, Moorepark, Fermoy, Ireland

CCAP: Culture Centre of Algae and Protozoa, Scottish Association for Marine Science, Dunstaffnage, Oban, Scotland

CLIB: Collection of Yeasts of Biotechnological Interest, Laboratory of Molecular and Cell Genetics, Thiverval-Grignon, France

INRA: Institut National de la Recherche Agronomique, Thiverval-Grignon, France
