# Supplementary material for: A Low Temperature Limit for Life on Earth
Source: PLoS One. 2013 Jun 19;8(6):e66207. doi: 10.1371/journal.pone.0066207 (PMC3686811; doi:10.1371/journal.pone.0066207)
Supplement: Table S2 — Summary statistics for rates of environmental temperature change (K hour−1). (DOCX) [file pone.0066207.s005.docx]

| Rate | n | Mean | Median | Q1 | Q3 | Min | Max |
| --- | --- | --- | --- | --- | --- | --- | --- |
| Mean | 8776 | 0.00065 | 0 | -0.4 | 0.4 | -5.5 | 5.4 |
| Maximum | 8776 | 0.0013 | -0.1 | -0.8 | 0.7 | -9.2 | 9.8 |
